# Environmental Mapping of *Paracoccidioides* spp. in Brazil Reveals New Clues into Genetic Diversity, Biogeography and Wild Host Association

**DOI:** 10.1371/journal.pntd.0004606

**Published:** 2016-04-05

**Authors:** Thales Domingos Arantes, Raquel Cordeiro Theodoro, Marcus de Melo Teixeira, Sandra de Moraes Gimenes Bosco, Eduardo Bagagli

**Affiliations:** 1 Departamento de Microbiologia e Imunologia, Instituto de Biociências de Botucatu, Universidade Estadual Paulista/UNESP, Botucatu, Brazil; 2 Instituto de Medicina Tropical—IMT/RN, Universidade Federal do Rio Grande do Norte/UFRN, Natal, Brazil; 3 Departamento de Biologia Celular e Genética, Centro de Biociências, Universidade Federal do Rio Grande do Norte/UFRN, Natal, Brazil; 4 Departamento de Biologia Celular, Universidade de Brasília–UnB, Brasília, Brazil; 5 Northern Arizona Center for Valley Fever Research, Translational Genomics Research Institute–Tgen North, Flagstaff, Arizona, United States of America; University of Tennessee, UNITED STATES

## Abstract

**Background:**

*Paracoccidioides brasiliensis* and *Paracoccidioides lutzii* are the etiological agents of Paracoccidioidomycosis (PCM), and are easily isolated from human patients. However, due to human migration and a long latency period, clinical isolates do not reflect the spatial distribution of these pathogens. Molecular detection of *P*. *brasiliensis* and *P*. *lutzii* from soil, as well as their isolation from wild animals such as armadillos, are important for monitoring their environmental and geographical distribution. This study aimed to detect and, for the first time, evaluate the genetic diversity of *P*. *brasiliensis* and *P*. *lutzii* for Paracoccidioidomycosis in endemic and non-endemic areas of the environment, by using Nested PCR and *in situ* hybridization techniques.

**Methods/Principal Findings:**

Aerosol (n = 16) and soil (n = 34) samples from armadillo burrows, as well as armadillos (n = 7) were collected in endemic and non-endemic areas of PCM in the Southeastern, Midwestern and Northern regions of Brazil. Both *P*. *brasiliensis* and *P*. *lutzii* were detected in soil (67.5%) and aerosols (81%) by PCR of *Internal Transcribed Spacer* (ITS) region (60%), and also by *in situ* hybridization (83%). Fungal isolation from armadillo tissues was not possible. Sequences from both species of *P*. *brasiliensis* and *P*. *lutzii* were detected in all regions. In addition, we identified genetic *Paracoccidioides* variants in soil and aerosol samples which have never been reported before in clinical or armadillo samples, suggesting greater genetic variability in the environment than in vertebrate hosts.

**Conclusions/Significance:**

Data may reflect the actual occurrence of *Paracoccidioides* species in their saprobic habitat, despite their absence/non-detection in seven armadillos evaluated in regions with high prevalence of PCM infection by *P*. *lutzii*. These results may indicate a possible ecological difference between *P*. *brasiliensis* and *P*. *lutzii* concerning their wild hosts.

## Author Summary

*Paracoccidioides brasiliensis* and *Paracoccidioides lutzii* are the fungal species responsible for one of the most important mycoses of Latin America, Paracoccidioidomycosis (PCM). These fungi can grow in soil from forests, deforested areas, sugarcane, coffee, and rice plantations, as well as pasturelands, and they are strongly associated to armadillo burrows, which can explain their frequent isolation from this mammal’s tissues. The environmental detection of these pathogens in endemic and non-endemic areas of PCM is important for mapping risk areas, as well as for understanding the infection ability and clinical manifestations of these fungi. These pieces of information are not provided by isolates obtained from human patients, because these fungi have a long latency period and the human host can migrate, leading to a misinterpretation of the actual geographic distribution of these pathogens. By using two different molecular methodologies (Nested PCR and in situ fluorescence), we detected both species of *P*. *brasiliensis* and *P*. *lutzii* in soil and in aerosol samples, even in areas where PCM is only associated to one of these two species. These data might indicate different habitat maintenance strategies between the species, which means that the infection ability may change according to the climatic and soil conditions. Despite contributing new information about the ecology of these important fungal pathogens, our molecular approach for the environmental detection of *Paracoccidioides* species may also be applied for their detection and differentiation in clinical samples, improving the diagnosis of this important systemic mycosis.

## Introduction

The study of biological and ecological aspects of *Paracoccidioides brasiliensis* [1,2] and *Paracoccidioides lutzii* [3] has been developed by several research groups in recent years. In particular, investigators have been seeking to isolate and/or detect those pathogens in clinical and environmental samples in order to obtain more in-depth data on the ecological factors that determine their geographical distribution. Both species cause paracoccidioidomycosis (PCM), the most prevalent systemic mycosis in Latin America, which can be acquired by non-immunocompromised hosts inhabiting and/or working mostly in endemic rural areas in South and Central Americas [4,5]. The disease is acquired by soil aerosolization and inhaling of infectious particles of the fungi [5]. It is known that *Paracoccidioides* spp. has its habitat (physical and geographical distribution site) located in the soil, but its ecological niche (sum of all interactions of the microorganism with the biotic and abiotic factors of the environment) has not been properly determined, demanding more environmental studies [4–6].

Studies on *Paracoccidioides* species distribution have been focused mainly on its isolation from human patients and scarcely from environment. Wild and domestic animals have been addressed due to the difficulties to retrieve the fungus from its habitat in laboratory conditions. The few cases of environmental pathogen isolation were from soil, foliage, dog food, bats and penguin feces, almost serendipitously, with little or no repeatability [4,7–10]. The frequent isolation of *Paracoccidioides brasiliensis* from armadillos, an animal whose home range is very limited, makes this mammal an excellent environmental source for mapping the geographic distribution of the fungus [5,11].

The difficulties faced by environmental studies limits the understanding about the ecology and the real distribution of this genus in endemic and non-endemic areas of PCM. For instance, the majority of the *Paracoccidioides* isolates used for molecular typing are from clinical specimens, which can be influenced by factors such as human migration and the long latency period of this mycosis, which may be greater than one or two decades [12,13], so that it is very hard to specify the exact local of infection and the occurrence of each cryptic species in the endemic and non-endemic regions where the patients are from. Identification of risk areas associated with the different species and/or genotypes can also contribute to a better understanding of *Paracoccidioides* biogeography and also to help the clinical procedures for PCM diagnosis and treatment [11,14]. However, little has been done given its methodological difficulties. In this study, a new approach based on an *in situ* hybridization technique with species-specific DNA probes, as well as the previously established molecular technique of Nested PCR [15,16], were carried out in order to detect the pathogen in different environmental samples, such as soil, aerosol and armadillo tissues. In this study, we expand the collection sites to include new areas of Southeastern, Midwestern and Northern Brazil (prevalent areas of *P*. *lutzii* infection). The main objective was to search for new ecological and biogeographical information about these fungi, in order to have a better profile of *P*. *brasiliensis* and *P*. *lutzii* distribution and dynamics in nature.

## Materials and Methods

### Ethics statement

Capturing of the armadillos was authorized by SISBIO-IBAMA for all of the Brazilian territory (IBAMA—30585–1 and IBAMA—37333–2), and the Animal Ethics Committee from Biosciences Institute of Botucatu—UNESP (protocol number 528) also approved the procedures.

### Selection of the study areas and environmental samples collection

**Fig 1:**
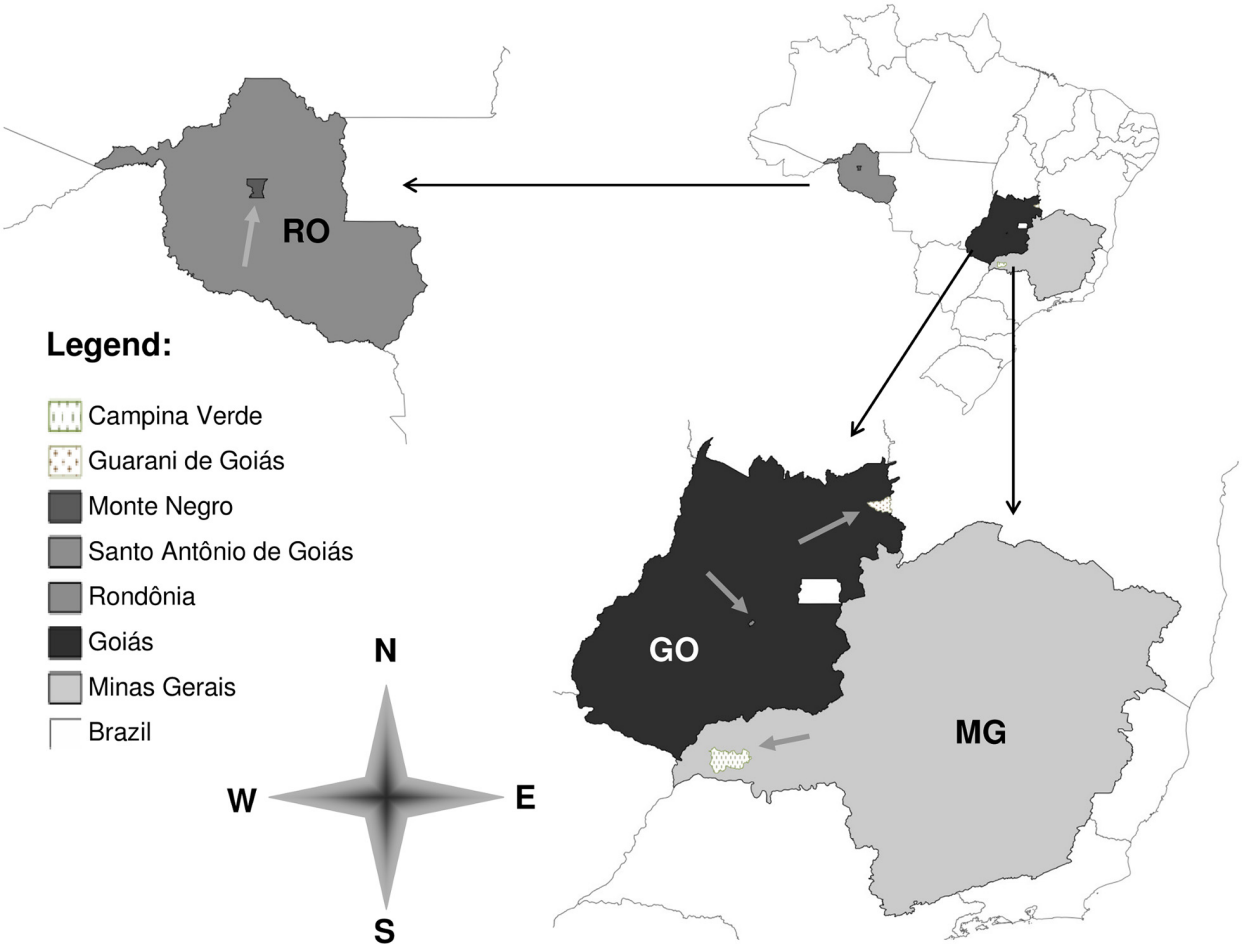
Collection areas of environmental samples (soil, aerosol and animals) in Brazil. The evaluated states (RO, MG and GO) are highlighted in different colors and the municipalities of sampling in different traces indicated with arrows.

Environmental samples from Rondônia (RO), Goiás (GO) and Minas Gerais (MG) states of Brazil, which have been poorly or never sampled, were selected for the molecular detection of *Paracoccidioides* spp. [17–19]. Samples were obtained by collecting aerosol and soil from armadillo burrows, as well as the animal specimen (*Dasypus novemcinctus)*. The distribution of collection areas along the Brazilian map are shown in Fig 1, highlighting the assessed municipalities: Monte-Negro (RO), Santo Antônio de Goiás and Guarani de Goiás (GO) and Campina Verde (MG). Georeferenced sites of the armadillo burrows in the collection areas are listed in Table 1.

**Table 1:** Georeferenced sites for the field work (soil, aerosols and armadillo collection).

ID	Description/Location	(S) Reference	(WO) Reference
**WP01**	Trail/SAG	16°29’52,7”	49°17’25,0”
**WP02**	Burrow/SAG	16°29’53,4”	49°17’25,9”
**WP03**	Burrow/SAG	16°29’10,3”	49°17’44,1”
**WP04**	Burrow/SAG	16°29’09,7”	49°17’44,0”
**WP05**	Burrow/SAG	16°29’37,5”	49°17’27,3”
**WP06**	Burrow/GDG	13°56’53,9”	46°29’14,6”
**WP07**	Burrow/GDG	13°51’42,6”	46°31’05,2”
**WP08**	Burrow/GDG	13°51’43,1”	46°31’05,4”
**WP09**	Burrow/GDG	13°51’43,3”	46°31’05,2”
**WP10**	Burrow/GDG	13°51’43,9”	46°31’04,1”
**WP11**	Embrapa/SAG	16°30’18,1”	49°16’52,0”
**WP12**	House/Posse-GO	14°05’12,0”	46°21’47,2”
**WP13**	House/GDG	14°51’40,8”	46°48’10,5”
**WP14**	House/CV	19°39’48,9”	49°22’07,7”
**WP15**	Burrow/CV	19°40’00,6”	49°22’03,4”
**WP16**	Burrow/CV	19°39’59,8”	49°22’01,5”
**WP17**	Burrow/CV	19°39’59,1”	49°22’02,4”
**WP18**	Burrow/CV	19°39’59,5”	49°22’02,4”
**WP19**	Burrow/CV	19°39’54,1”	49°21’54,8”
**WP20**	Burrow/CV	19°39’57,9”	49°21’48,5”
**WP21**	Burrow/CV	19°39’57,4”	49°21’47,9”
**WP22**	Burrow/CV	19°39’57,5”	49°21’50,6”
**WP23**	Burrow/MN	10°18’46,3”	63°16’57,0”
**WP24**	Burrow/MN	10°18’56,2”	63°16’56,0”
**WP25**	Burrow/MN	10°17’23,3”	63°16’33,3”
**WP26**	Burrow/MN	10°17’19,5”	63°16’57,1”
**WP27**	Burrow/MN	10°14’41,6”	63°18’14,3”
**WP28**	Burrow/MN	10°14’05,9”	63°18’16,4”
**WP29**	Burrow/MN	10°09’09,6”	63°16’18,1”
**WP30**	Burrow/MN	10°09’09,1”	63°16’17,8”

**Legend:** Santo Antônio de Goiás (SAG); Guarani de Goiás (GDG); Campina Verde (CV) and Monte Negro (MN); (S)–South; (WO)—East-West; For Armadillo: the WayPoints (WP) 13, 14 and 23 represent areas around the collection sites for captured animals in RO, MG and GO states.

### Collection of soil and armadillo specimen samples

About 50g of soil was collected from the armadillo burrows by using an iron spatula. The samples were carefully collected so that the burrows were not destroyed. The soil was placed in 50 mL sterile universal bottles, sealed, identified, stored at room temperature, for up to fifteen days, and processed for DNA extraction, accordingly with our previously experience [15,16]. During the collection procedure, the instruments were decontaminated with 70% v/v ethanol solutions in order to avoid cross contamination of samples from one location to another [15,16]. For soil samplings, the minimum established number was 10 samples for burrows and trails at each site studied. In addition to the collected samples, a soil called "Dark Earth" from the occidental Amazon region (which is an anthropogenic soil and rich in organic matter) was kindly provided by the Center for Nuclear Energy Research Group in Agriculture (CENA)—USP, under the responsibility of PhD. Siu Mui Tsai. These soil samples were processed with the same methods described above (storage and DNA extraction).

For the capture of armadillos in the field, track traps were used and we were assisted by local hunters in order to identify areas with high animal activity. Track traps were placed in the armadillo’s trails next to those burrows for which recent animal activity had been detected. In addition to the track traps, active capture took place in the evening, the period of the animal’s highest activity.

Captured animals were placed in containers with fresh and dry straw to better accommodate them during transportation until the euthanasia procedure in the laboratory. The number of animals varied according to the season (rain or dry), as well as to the difficulties for finding specimens and transporting them to the laboratory. The euthanasia for the evaluated animals in this study was performed by subcutaneous administration of Zoletyl 50 (0.2 mL/kg/IV, Virbac), followed by cardiac puncture and administration of potassium iodine to ensure the animal's death. Spleen, liver and mesenteric lymph nodes were removed and placed in sterile plates and soaked in alcohol 70% v/v for a brief cleaning followed by saline solution (0,9% w/v). Small fragments (1-2mm) of the organs were then placed in a Mycosel Agar culture medium (Difco) supplemented with 50 μg/mL Gentamicin and incubated at 35°C during 45 days [11,16]. After the evaluation, these plates were properly sterilized and discarded.

### Aerosol samples

Air sampler model Cyclone 251 BC, developed by the Centers for Diseases Control—CDC (Morgantown, WV, USA) and certified by the National Institute for Occupational Safety and Health (NIOSH) coupled to vacuum pumps type 224-44XR Model SKC Universal Pumps was used for air sampling [20]. An air sampler was placed next to the armadillo burrows as well as in the areas where the animals had recently removed the soil searching for food. The vacuum pump has a rechargeable battery life up to 24 hours of operation at maximum flow, thereby facilitating the procedures for collection in remote locations without any power sources. Aerosol and soil samples were collected in the states of GO and MG during the dry/warm season, and in RO state during the cold/rainy season. Each aerosol collection was performed in a minimum period of 60 minutes with a flow rate of 3500 mL/min. At least four samples were collected at each site.

### DNA extraction and PCR/nested PCR reactions

Each soil sample was subjected to total DNA extraction in triplicate, using the commercial kit PowerSoil DNA Isolation Kit—MO BIO Laboratories, Inc. Total DNA was resuspended in 100 μl of Nuclease free water and quantified in NanoVue spectrophotometer equipment (GE Healthcare). Fifty microliters of each one of the three replicates were mixed in a single 1.5 mL tube and concentrated to a final volume of 30μl in a concentrator (Eppendorf) and then quantified again to confirm the new concentration of each sample. The aerosol samples were directly used for PCR without any previous DNA extraction by washing the tubes from cyclonic sampler with the PCR reaction mix.

The PCRs (Polymerase Chain Reaction) were carried out with ITS-4/5 primers for rRNA universal fungal region ITS1-5.8S-ITS2 (*Internal Transcribed spacer*) [21]. A Nested PCR was performed with the product of the first amplification using specific primers for the *Paracoccidioides* genus, annealing in the ITS-1 and ITS-2 regions, named PbITS-E (5’GAGCTTTGACGTCTGAGACC3’) and PbITS-T (5’GTATCCCTACCTGATCCGAG3’) [16]. Both PCR mixes were prepared using 12.5 μl of Nuclease Free Water (Sigma), 0.5 μl of 0.2 mM dNTP mix, 5.0 μl of 5X GC buffer, 2.5 μl of 30% DMSO, 0.625 μl of each primer at 20 μM and 0.25 μl of 1000 units/μl Taq Phusion DNA Polymerase (ThermoFisher) for each reaction with 23.0 μl of PCR mix for 2.0 μl from a DNA soil sample of approximately 15 ng/μl. Twenty five microliters of PCR reaction was performed for aerosol samples, these mixes were prepared from 100.0 μl of reaction mix used for washing the aerosol collection tubes, so that four PCRs were carried out for each aerosol sample.

The thermal cycling conditions for the first PCR were: an initial denaturation at 98°C for 30 seconds and 39 cycles of denaturation at 98°C for 10 seconds, followed by an annealing step at 55°C for 45 seconds, and extension at 72°C for 45 seconds, after that a final extension at 72°C for 10 minutes was applied. For aerosol samples, the first step of denaturation was longer (5 minutes) than the one applied to the DNA soil samples, in order to break the spores and other fungal structures, releasing the genetic material in the PCR mix. For Nested PCR, the annealing step was adjusted to 58°C.

After the PCR and Nested PCR reactions, PCR products were analyzed by electrophoresis in a 1.5% w/v agarose gel. The bands around 450bp (*Paracoccidioides* spp.) or that were best highlighted in the gel were cut out, purified by using the commercial Kit (GE illustrates GFX PCR DNA and Gel Band Purification) and quantified as described above. Purified samples were sent to the Laboratory for Molecular Diagnosis of the Department of Microbiology and Immunology (UNESP, Botucatu/SP-Brazil) for automatic capillary sequencing in ABI 3500 DNA Analyzer (Applied Biosystems) equipment. For low concentration of PCR products, a new amplification reaction with the PbITS-E/T primers were performed (double PCR), followed by its purification and sequencing.

The obtained sequences were aligned to the reference sequences with the help of the MEGA 6.0 program [22] and compared to an online database (GenBank) [23], to verify their identity and phylogenetic clusterization with other deposited ITS sequences from *P*. *brasiliensis* and *P*. *lutzii*.

### Phylogenetic analysis

The initial sequences obtained from environmental amplicons were checked using the Sequencing Analysis software from ABI 3500 DNA Analyzer (Applied Biosystems) in order to improve the quality of sequences. For phylogenetic analysis and comparison of other *Paracoccidioides* DNAs, we used the deposited sequences under the following access numbers: EU870314; EU870315; EU870316; AY631235; EU118561; EU118560; EU118548; EU118554; EU118553; EU118549; EU118546; EU118547; EU118545; EU118543; EU118542 for *P*. *brasiliensis* and EU870298; EU870303; EU870306; EU870309; EU870310; EU870311; AF092903; EU870299 for *P*. *lutzii*. Only the sequences, herein obtained from environmental samples, presenting ≥ 97% of similarity to *Paracoccidioides* spp. sequences in *GenBank* (blastn analysis) [24] were considered for phylogenetic analysis in MEGA 6.0 software. Sequences previously generated from soil and aerosol samples from the Southeast part of Brazil [16] were included in this dataset, access numbers KP636439 to KP636474 (S1 Table). In addition, clinical strain sequences from *GenBank*, representing *P*. *lutzii* (Pb01—EU870297), *P*. *brasiliensis* species complex S1 (Pb18—AF322389), PS2 (Pb3—EU870315), and PS3 (AY631237) were included into the final dataset. Sequences were aligned with the ClustalW [25] algorithm implemented in the Bioedit software [26]. Retrieved alignments were manually inspected in order to avoid mispaired bases. Neighbor-Joining [27] and Maximum Likelihood trees were inferred in the MEGA 6 software [22] using the Jukes-Cantor [28] nucleotide substitution model. One thousand bootstrap replicates [29] were used to estimate the monophyletic clades support, and values were displayed next to the branches.

### Detection and differentiation of *P*. *brasiliensis* complex and *P*. *lutzii* species by *in situ* hybridization in environmental aerosol samples

**Fig 2:**
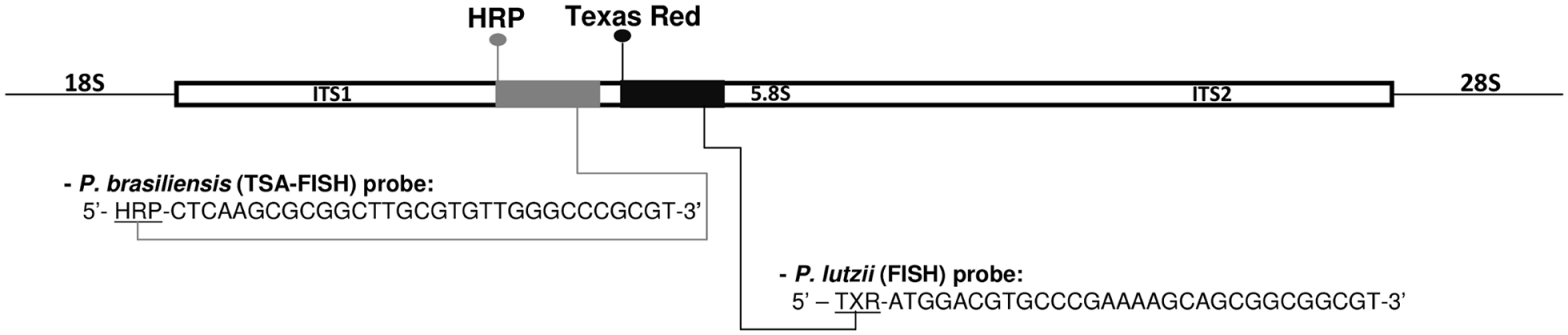
The ITS region with the location of oligo probes designed for the detection and differentiation of *P*. *brasiliensis* and *P*. *lutzii*, labeled with HRP and Texas Red, respectively.

Probes used in this study were commercially synthesized targeting the rRNA region of *P*. *brasiliensis* and *P*. *lutzii*, specifically for the ITS-1-5.8S-ITS-2 region [14,30,31]. Sixty ITS sequences from different *Paracoccidioides* isolates were aligned in order to select conserved regions within species, exclusive and different between *P*. *brasiliensis* and *P*. *lutzii* (Fig 2). The probes were differentially labeled on their 5’ end, with Horseradish Peroxidase (HRP) for *P*. *brasiliensis*, and Texas Red for *P*. *lutzii*, (Fig 2).

The use of this approach for detection of *Paracoccidioides* spp. as well as differentiation between the *P*. *brasiliensis* complex and *P*. *lutzii* species was previously standardized [32]. For those methods, each probe was tested against other Ajellomycetaceae fungi (*Histoplasma capsulatum*) and other clinically relevant ascomycetes (*Aspergillus flavus*; *Aspergillus fumigatus*; *Trichophyton mentagrophytes*), as negative controls for both probes (used in FISH and TSA-FISH techniques). Twelve aerosol samples were collected in armadillo burrows for *in situ* hybridization method: four from the state of GO, four from MG and four from RO.

The detection of *Paracoccidioides* spp. with DNA probes by the TSA-FISH method in aerosol samples was applied for greater sediment volume in the cyclone sampler tubes (stages). These samples from both stages of the cyclone sampler were fixed with 1.5 mL of 4% Paraformaldehyde plus 0.1 M Phosphate solution buffer. Series of 50%, 80% and 100% ethanol solutions were used to remove cell fixation solution and to dehydrate the cells, so that they have the ability to absorb the probes to be used in the hybridization step. After dehydration, 10 mL of pre-hybridization buffer [2.0 mL of ultra-pure water; 4.0 mL of 40% Formamide; 1.8 mL of 5 M NaCl; 200 μl of 1 M Tris (pH 7.5); 100 μl of 1% SDS; 2 mL of 10% Buffer Blocking Agent] were added to the samples for stabilization and improvement of their permeability. After this first step, cells were hybridized with probes at a final concentration of 50 ng/μl in hybridization buffer. After 16–17 hours of incubation at 42°C, the slides with fungal controls were washed with 50 mL of Washing Buffer [47.54 mL of ultra-pure water; 460 μl of 5 M NaCl, 500 μl of 0.5M EDTA, 500 μl of 1% SDS and 1 mL of 1 M Tris (pH 7.5)] for removal of non-specific binding probes. After washing, the slides were stabilized with 250 mL of TNT buffer [217.315 mL of ultra-pure water; 25 mL of 1 M Tris (pH 7.5); 7.5 mL of 5M NaCl and 0.185 mL of Tween 20].

After equilibrating and washing the slides with TNT buffer, 30 μl of TSA solution (TSA Plus PerkinElmer Kit) were added to each slide and incubated for 30 minutes in a humid dark chamber at room temperature. The slides were washed again, dried at room temperature, prepared with the addition of 4’,6-Diamidino-2-phenylindole dihydrochloride (DAPI) and covered with a cover slip to be observed under a fluorescence microscope. These slides were divided in two groups of four slides each: one was tested against *P*. *brasiliensis* probe and the other against *P*. *lutzii* probe. Two spare slides were used as controls during the hybridization phase for each of the two methods and probes used in this study.

## Results

### Animals evaluated in the collected areas

Seven armadillos were captured and evaluated, three from GO, three from RO and one from MG states. Information about the gender, weight and cultivated organ fragments are listed in Table 2.

**Table 2:** Armadillos (*Dasypus novemcinctus*), and their respective organ fragments, evaluated in the Brazilian states of Goiás, Minas Gerais and Rondônia.

ID Animal	Sex	Weight	Spleen (Plates)	Liver (Plates)	L. M. (Plates)	Total of Plates	Number of Fragments
**AGO1**	Male	5.5 kg	12	66	06	84	1,950
**AGO2**	Male	4.3kg	09	53	02	64	1,966
**AGO3**	Male	4.5kg	21	44	03	68	2,548
**ARO1**	Female	5.0kg	12	20	05	37	1,050
**ARO2**	Female	4.3kg	12	20	05	37	1,060
**ARO3**	Male	1.8kg	10	20	06	36	1,000
**AMG1**	Male	4.5kg	15	48	07	70	2,450
**Total of Organ Fragments**							**12,024**

**Legend:** AGO—Armadillos from Goiás; ARO—Armadillos from Rondônia; AMG—Armadillos from Minas Gerais; L.M.–mesenteric lymphnodes; Plates—Total number of culture plates assessed for each organ; Number of Fragments—total number of fragments from each organ that were cultured in Mycosel agar for each animal evaluated.

After 45 days of incubation, each plate was evaluated for fungal growth similar to *Paracoccidioides* spp. by micro-morphological analysis. All the colonies presented morphological structures of bacteria, despite the addition of antibiotics to the culture medium and no fungal structures were identified. After 45 days, the plates were then considered negative for *Paracoccidioides* spp. growth.

### Nested PCR, phylogenetic and haplotype analysis of soil and aerosol samples

Forty-four soil samples from armadillo burrows were obtained; 12 in GO, 10 in MG and 22 in RO. Twenty-eight aerosol samples from armadillo burrows were obtained; 10 in GO, 10 in MG and 08 in RO, 16 of these samples were set aside for molecular detection by Nested PCR and the rest of the 12 aerosol samples were used for *in situ* hybridization techniques. The sampled burrows in the areas of RO, GO and MG states were mostly located in deforested areas of pastures or in some riparian forest sites. Positive Nested PCR amplification for ITS region of *Paracoccidioides* spp. was observed in 67.5% of soil samples and in 81% of aerosol samples. No amplicons were observed for “Dark earth” soil samples. When compared to the *GenBank* database, sequences revealed SNPs specific for *P*. *lutzii* and/or *P*. *brasiliensis* in all the positive soil samples from RO, GO and MG. *P*. *lutzii* was found in aerosol samples in MG and GO states, while *P*. *brasiliensis* was only detected in GO (Table 3).

**Table 3:** Detection of *Paracoccidioides* spp. by Nested PCR and *in situ* hybridization techniques, both in soil and aerosol samples in the states of Goiás, Minas Gerais and Rondônia, Brazil.

	Nested PCR						*in situ* hybridization		
	Soil		Total # of collected samples	Aerosol		Total # of collected samples/ PCR Reactions*	Aerosol		Total # of samples
Location	*Pb*	*Pl*		*Pb*	*Pl*		*Pb*	*Pl*	
**Goiás**	**0**	**9 (75%)**	**12**	**4 (16.5%)**	**0**	**6/24**	**4 (100%)**	**2 (50%)**	**4**
**Minas Gerais**	**0**	**0**	**10**	**0**	**9(37.5%)**	**6/24**	**2 (50%)**	**0**	**4**
**Rondônia**	**0**	**14 (64%)**	**22**	**0**	**0**	**4/16**	**1 (25%)**	**1 (25%)**	**4**
	**Positivity: 67.5%**			**Positivity: 81%**			**Positivity: 83%**		

**Legend: *Pb***–*Paracoccidioides brasiliensis*; ***Pl***–*Paracoccidioides lutzii*;

*—Total # of collected samples/ PCR Reactions shows the number of total samples collected; for each sample, the PCR mix was divided in 4 separated reactions. The positivity of Nested PCR for the aerosol samples is based on the number of positive amplification out of the total of PCR reactions.

**Fig 3:**
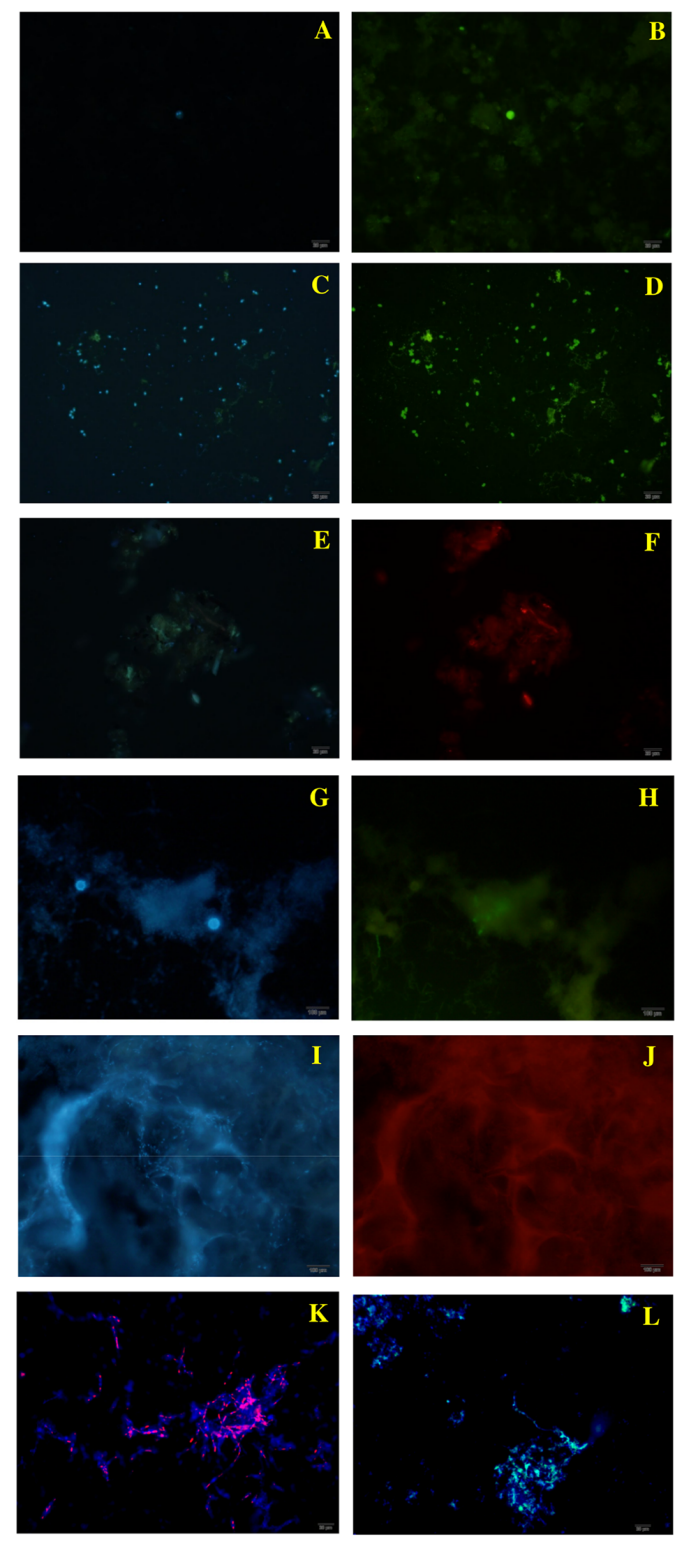
**A)** Molecular Phylogenetic Analysis by of ITS locus revealed by Maximum Likelihood and Neighbor Joining methods, using the Jukes-Cantor model parameters with range correction. Replication percentages on the tree are grouped in the bootstrap test (1000 replicates) and shown next to the branches. The sequences related to environmental samples are identified by acronyms SO_GO (Soil of Goiás) and AR_GO (Aerosol Goiás), AR_MG (Aerosol Minas Gerais) and SO_RO (Soil of Rondônia). **B)** Median-joining network showing the unique haplotypes of the Soil Clades I and II. Circles are proportional to haplotype frequency and numbers of mutations are represented by black dots. Red circles represent hypothetical missing intermediates (median vectors).

Phylogenetic analyses were carried out using a total of 36 sequences obtained from the current study and 11 sequences collected from the GenBank were added to the final dataset. The majority of the soil and aerosol samples clusterized within *P*. *lutzii* species (27 out of 36 –Fig 3A). *P*. *lutzii* was detected in GO, RO and MG states, as well as in São Paulo (SP) as previously reported [16]. All these *P*. *lutzii* sequences were displayed in a single haplotype together with the referenced clinical strain Pb01 (Fig 3B). Solely 4 samples (AR_GO1, AR_GO19, AR_GO11 and AR_GO2D) were clustered within *P*. *brasiliensis*, all obtained from aerosol samples collected in GO state (Fig 3A). However, only the AR_GO1 and AR_GO19 samples are clustered with the clinical strains Pb3, Pb18 and ATCC60855 in unique haplotype representing clinical isolates from the *P*. *brasiliensis* species complex S1, PS2 and PS3 (Fig 3B).

The phylogenetic distribution and haplotypic network analysis revealed higher genetic variation in the environmental samples than reported so far for *Paracoccidioides* clinical samples. The samples AR_GO11 and AR_GO2D are disposed to polytomic branches within *P*. *brasiliensis* and constitute single haplotypes in the network (Fig 3). Moreover, two high supported clades were observed as being closely related to *P*. *lutzii* clinical/environmental samples (Soil clades). The soil clade I is composed of the soil samples SO_GO10 and SO_GO19 from GO state, while the soil clade II is composed of soil samples SO_RO11 and SO_RO12 from RO state. In addition, the AR_MG16 sample collected from aerosol in MG also appears to be a genetic variant from *P*. *lutzii*, fallen into a paraphyletic branch in the tree (Fig 3A).

### Detection and differentiation of *P*. *brasiliensis* and *P*. *lutzii* in aerosol samples by *in situ* hybridization

The detection and differentiation of *Paracoccidioides* spp. in aerosol samples was performed after validation of FISH and TSA-FISH in *P*. *brasiliensis* and *P*. *lutzii* in culture cells. Specificity and sensitivity control tests were applied for validating the positive detection in environmental samples [32]. The probes tested (conjugated with HRP and Texas Red) did not hybridize with the negative controls. Both *P*. *brasiliensis* and *P*. *lutzii* cells under culture conditions show specific nuclear staining, which merge with DAPI.

For the *in situ* hybridization, the aerosol samples with higher sediment in the tubes were obtained in dry areas (GO and MG) where the burrow soil was easily aerosolized. For the RO state samples, which were obtained during an intense rainy season, the pellets were less visible to the naked eye.

**Fig 4:**
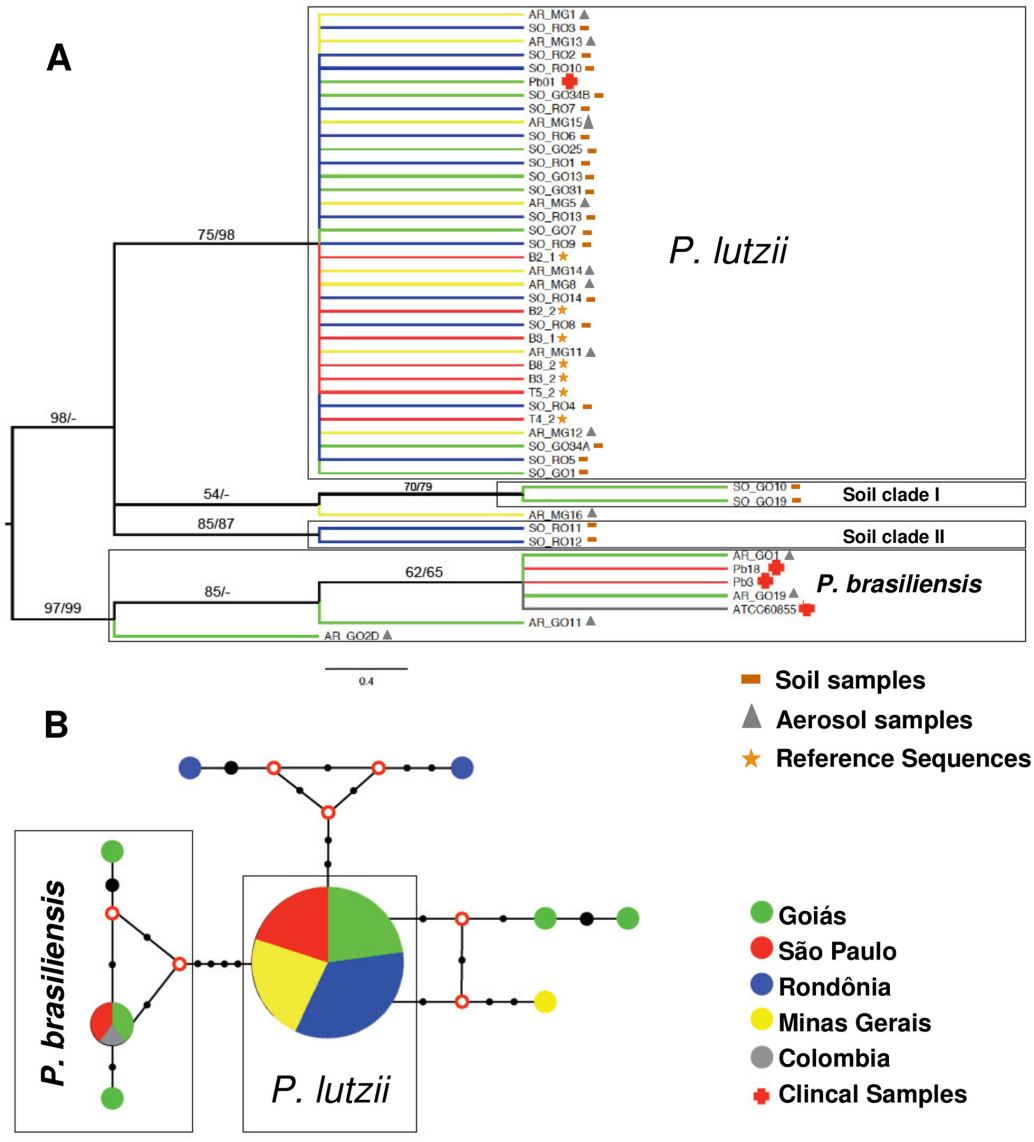
Fungal structures (400X) visualized by FISH and TSA-FISH techniques for aerosol samples and controls. **A:** aerosol samples from Goiás with DAPI. **B:** aerosol samples from Goiás with *P*. *brasiliensis* probe. **C:** aerosol sample from Rondônia, with DAPI. **D:** aerosol samples from Rondônia with *P*. *brasiliensis* probes. **E:** aerosol sample from Goiás with DAPI. **F:** aerosol sample from Goiás with *P*. *lutzii* probe. **G and I:** *Histoplasma capsulatum* with DAPI. **H:** *Histoplasma capsulatum* with *P*. *brasiliensis* probe (specificity control). **J:** *Histoplasma capsulatum* with *P*. *lutzii* probe (specificity control). **K:** isolate Pb01 (*P*. *lutzii*) with *P*. *lutzii* probe (positive control). **L:** isolate T16B1 (*P*. *brasiliensis*) with *P*. *brasiliensis* probe. The probe for *P*. *brasiliensis* is conjugated with Horseradish Peroxidase/HRP and *P*. *lutzii* probe is labeled with Texas Red/TXR, and genetic material is labeled with DAPI.

From the 72 slides prepared for the *in situ* hybridization, positive hybridization occurred in 36 (50% of the slides), revealing that ten out of 12 air samples from the armadillo burrows (83%) were positive for *Paracoccidioides* spp. (Table 3). The *in situ* hybridization experiments using specific probes for *P*. *brasiliensis* and *P*. *lutzii* revealed the presence of both species in GO and RO states (Fig 4A, 4B, 4E and 4F), and only *P*. *brasiliensis* species in MG state (Fig 4).

**Fig 5:**
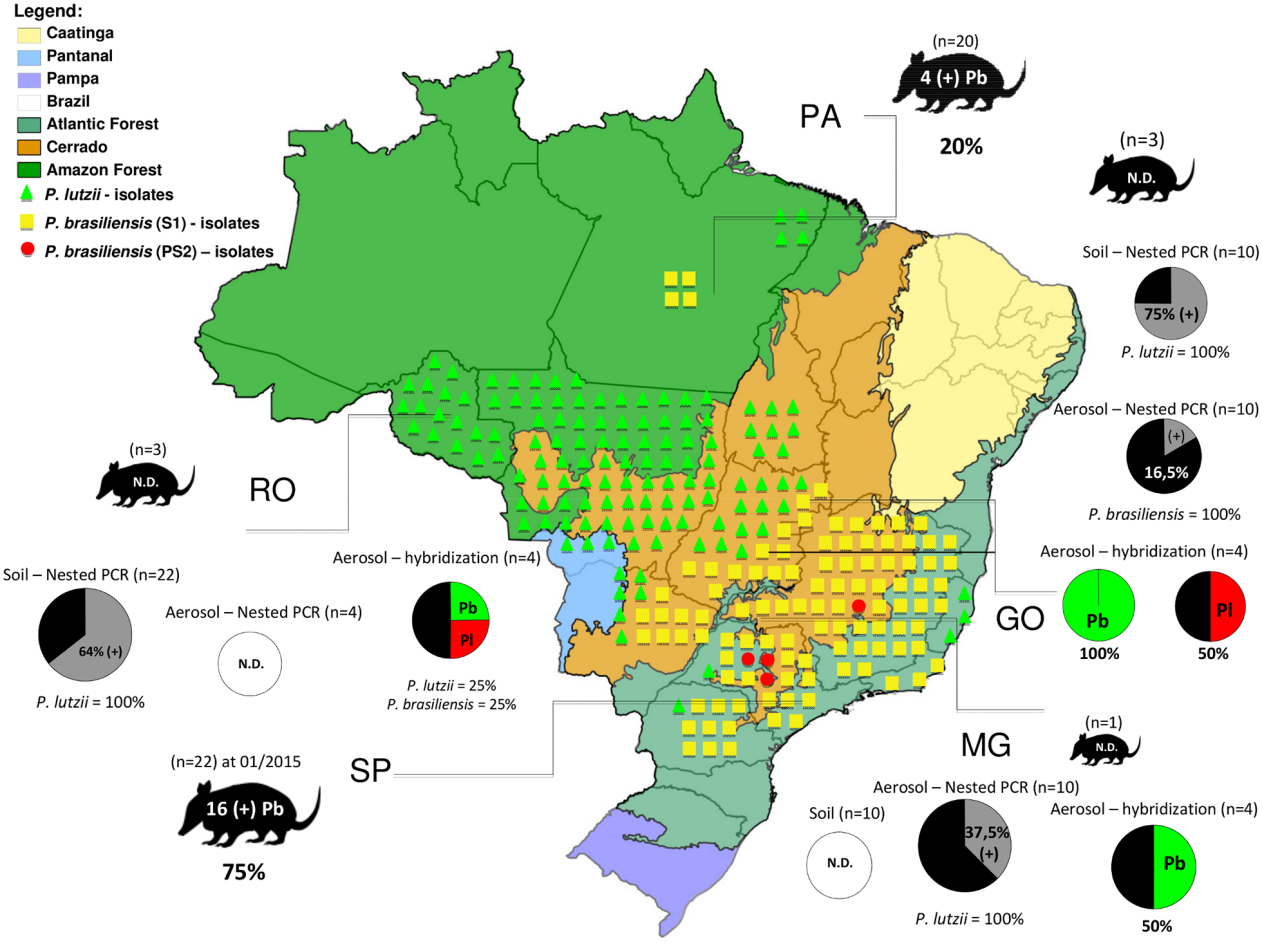
Brazilian sites and biomes where environmental detection (from soil, aerosol and armadillos) of *Paracoccidioides* spp. has been carried out. The collection areas encompass the states of Minas Gerais (MG), Goiás (GO) and Rondônia (RO) states, to São Paulo (SP) and Pará (PA). Circles outside of the map indicate the percentage of positivity in each area for *P*. *brasiliensis* (green) and *P*. *lutzii* (red) by *in situ* hybridization for aerosol samples. The circles outside of the map in black and grey indicate the percentage of positivity in each area for *P*. *brasiliensis* and *P*. *lutzii* by Nested PCR for soil and aerosol samples. The white circle indicates the negative detection in the evaluated areas (N.D. = Not detected). The armadillos indicate the number of collected animals and the positivity for isolation of *Paracoccidioides brasiliensis* (Pb) in each location of our study (in RO, GO and MG) and from previous works in SP [16] and PA [33]. The triangles (green), squares (yellow) and circles (red) show the distribution of the clinical isolates for cryptic species (S1, PS2 and *P*. *lutzii*) in previous studies [14,31].

Fig 5 summarizes the current data on *Paracoccidioides* spp. detection in soil and aerosol samples by the different methodologies applied so far, including the current results and those previously obtained [16].

## Discussion

Soil and aerosol samples have shown to be excellent sources for mapping the fungus in endemic areas of PCM by molecular methods, since they are easy to obtain and handle in a laboratory, therefore corroborating previous studies of our group [15,16]. Both soil and aerosol samples were positive for the environmental detection of *Paracoccidioides* spp. DNA in the sampled areas, revealing the ubiquitous distribution of these pathogens in the Brazilian territory. Aerosol samples were collected in a smaller number compared with the soil samples, due to the methodological difficulties in the field and weather conditions of each sampled location. The soil collection methodology is faster and easier to perform than the aerosol sampling, which requires more field effort to become representative. On the other hand, the aerosol sampling mimics the fungal dispersion mechanism by which rural workers become infected with the mycelia and/or conidia particles. Currently, soil from endemic or non-endemic areas for PCM may contain fungal cells with infectious potential for human and/or animal population, although the course of infections can vary according to the biotic and abiotic factors of the environment. It has been stated that agricultural activities are the activities that most favor PCM infection in humans [34,35]. The aerosol samples are also methodologically simple to work with in the laboratory because DNA extraction is not required.

Molecular detection in soil samples was positive in two of the sampled areas (RO and GO), but it was negative in MG. The molecular detection of *Paracoccidioides* in RO soil showed higher amplification rate as visualized in the agarose gel electrophoresis (S1 Fig), probably because of the rainy conditions during the collection, which according to Barrozo et al., 2009 and 2010 [34,35], may favor fungus maintenance in soil, as well as its dispersion, causing increased incidence of PCM. Positive samples were also obtained from northeastern Goiás, a warm and dry area, which might indicate some fungal resistance to adverse conditions.

This work presented the first standardization of *in situ* hybridization techniques for environmental search of *Paracoccidioides* spp., (HRP-probe/TSA-FISH for *P*. *brasiliensis* and Texas Red-probe/FISH for *P*. *lutzii*), making its detection possible in aerosol samples from the three locations studied (RO, MG and GO). This new approach showed sensitivity and specificity rates similar to the well-established Nested PCR technique. The advantage of *in situ* hybridization is the visualization of infective fungal structures of *Paracoccidioides* spp. directly in the environmental samples.

The detection rate of *Paracoccidioides* spp. in soil and aerosol samples in GO, MG and RO was lower than the detection rate in endemic areas of PCM. In most cases, the fungus was detected in locations whose air humidity and temperature conditions were very similar to those found in endemic areas of PCM. However, new distribution nuances of *P*. *brasiliensis* and *P*. *lutzii* were revealed in our study, including a remarkable resistance to adverse environmental conditions, so that the spatial distribution of the *Paracoccidioides* species may be larger than previously defined based mainly on clinical isolates [30,31,36].

Both growth and dispersion of this pathogen seems to be greatly influenced by the climate. While high moisture levels increase fungal growth and maintenance in soil, a brief drought period dries the most superficial layer of the soil, making the dispersion of aerosols (mycelia particles/conidia and other microorganisms) easy and intense [37]. This was already observed in *Coccidioides* spp. [38,39] and could explain the negative detection of *Paracoccidioides* spp. in aerosol samples from RO, which faced one of the most severe rainy periods of the last few years [40]. This explains the higher positive molecular detection of *Paracoccidioides* spp. by Nested PCR in soil samples than in aerosol samples from RO, where the collection was carried out during the raining season. Therefore, different from soil samples, which directly demonstrate the presence of the fungus, and aerosol samples also reflect the spread of the fungus in the environment, and therefore its infective potential is extremely important in the epidemiological study of *Paracoccidioides* spp., corroborating the growth and blow theory which reflects the crescent number of cases after rainy seasons in endemic areas [34,35] and the great recent number of new PCM cases in areas of North Brazil, as RO state [19].

In RO and GO, the detection of *Paracoccidioides* spp. was positive mostly in deforested pastures and in some riparian forest sites. In fact, such areas with increased agricultural activity present the greatest incidence of PCM cases in these states [17–19]. Deforestation of preserved areas exposes the soil and naturally or deliberately changes its chemical conformation, which can favor the infection of rural workers or other people living in these areas, leading to the emergence of PCM. Our studies indicate that *P*. *lutzii* and *P*. *brasiliensis* is often found in soil and aerosol samples in all four of the sampled regions. For this reason, we hypothesized that the geographic distribution of PCM caused by different *Paracoccidioides* species may be associated to the capacity of each fungal species to produce infective propagule in the current environmental conditions, which includes soil management for local agriculture activity (sugar-cane, coffee and cattle breeding). For instance, despite the environmental molecular detection of *P*. *brasiliensis* in Goiás and *P*. *lutzii* in Minas Gerais, the majority of PCM cases in these states are caused by *P*. *lutzii* and *P*. *brasiliensis*, respectively.

Considering the difficulty for animal capture and transportation, a reasonable number of armadillos for each sampled area were obtained. All armadillos evaluated in this study were negative for culture isolation of *Paracoccidioides* spp. However, molecular detection in soil and aerosol samples indicated the presence of *Paracoccidioides* spp. in these areas. This negative result for the non-endemic areas, where these animals were collected (northeastern GO and MG), may point to the relationship of environmental conditions and the possibility of infection and/or disease in these animals, and probably in humans too. On the other hand, the negative isolation in armadillos from the endemic area (RO) is more intriguing. In this area, most of the sequences detected showed high similarity to *P*. *lutzii* species (in all positive soil samples by Nested PCR, and in 50% of the positive aerosol samples by *in situ* hybridization). This may indicate that the relationship between armadillo and *P*. *lutzii* is different from the well-known interaction between armadillo and *P*. *brasiliensis*, which could have resulted in a speciation process (*P*. *brasiliensis* complex X *P*. *lutzii*) driven by hosts. *P*. *brasiliensis* S1 and PS2 species (São Paulo state/Brazil) and PS3 (Colombia) being highly recovered from the armadillos, and *D*. *novemcinctus* and *Cabassous centralis* [11,41] in endemic areas, while no *P*. *lutzii* has been isolated from these animals yet.

According to the phylogenetic analysis, there was a prevalence of sequences belonging to the *P*. *lutzii* species in all evaluated areas, which reflect the distribution pattern of clinical isolates observed in recent works [14,16,18]. Despite the negative isolation of *Paracoccidioides* spp. from armadillos, data from molecular detection of these pathogens in soil and/or aerosol samples may be useful for delineating the geographic distribution of *Paracoccidioides* spp.

Herein for the first time, environmental genetic variants were reported for *Paracoccidioides* genus (Fig 3). Phylogenetic and haplotype data revealed the presence of two well-supported clades in environmental soil samples, one from GO (Soil Clade I) and the other from RO (Soil Clade II). The sample AR_MG16 collected from aerosol in MG also appears to be a genetic variant from *P*. *lutzii*, fallen into a paraphyletic branch in a tree. It’s worth noting that all environmental samples that clustered apart from the clinical referenced strains of both *P*. *lutzii* and *P*. *brasiliensis* are displayed in single haplotypes, reinforcing a higher diversity of the *Paracoccidioides* ssp. in the environment than in human hosts. This discrepancy shows that part of the environmental genotypes of *Paracoccidioides* spp. may not be able to infect and/or cause PCM.

It is interesting to note that although Central-Western Brazil presents a prevalence of PCM caused by *P*. *lutzii*, *P*. *brasiliensis* was also detected in these areas in this work and in previous studies [30,31,42]. This observation could indicate different patterns of sporulation depending on soil constitution and weather, so that the conidia production/release of *P*. *brasiliensis* and *P*. *lutzii* could be different in distinct areas, explaining the current distribution pattern of PCM caused by both species. Previous studies have already pointed out differences in sporulation (concerning conidia amount and morphology) among the different cryptic species: S1 isolates produce a higher conidia amount compared to other *P*. *brasiliensis* and *P*. *lutzii* species. This observation may explain the higher isolation of S1 species (from humans and armadillos) in endemic areas for PCM caused by *P*. *brasiliensis* [42,43].

Molecular epidemiology studies of fungal pathogens are extremely important, since the genetic variability may reflect the existence of cryptic species, can have a geographic pattern and also be related to different clinical manifestations and antifungal drug response. For that reason, many efforts have been made to study the geographic limits of the different genotypes of some fungal pathogens, such as *Histoplasma capsulatum*, a complex of at least seven distinct clades, whose variability has been addressed by MLST (Multi-locus sequencing type), as well as ITS and PRP8 intein genes as molecular markers [44–46]. Cryptic speciation event has also been recently described for another Ajellomycetacea member, *Blastomyces dermatitidis*, which is now considered two species, *B*. *dermatitidis* and *Blastomyces gilchristii*. Like *P*. *brasiliensis* and *P*. *lutzii*, these *Blastomyces* species seem to be sympatric in some of their distribution ranges [47].

Despite that ITS region is widely used as fungal barcoding [48], most studies on cryptic speciation use the multi-locus sequencing approach. That is because ITS is, in some cases, useless to distinguish among very close cryptic species, such as those from the *P*. *brasiliensis* complex [31]. However *P*. *brasiliensis* and *P*. *lutzii* are clearly distinguished by ITS sequencing. Thus, our findings in fact confirm the coexistence of the *P*. *lutzii* and *P*. *brasiliensis* species (S1 and PS2 species) in the environment, even though the potential for human infection seems to be different for both species, depending on the region.

This study presents important data regarding the eco-epidemiology of *Paracoccidioides* species, as well as their actual distribution over the Brazilian map. In addition, new environmental genetic variants of these pathogens should notify us about the actual scenario of the *Paracoccidioides* species diversity. Our results also pointed out the possible differential pathogen versus wild host interaction among *Paracoccidioides* species, generating new hypotheses and new work issues to be tested and studied by the scientific community. Thus, further studies based on data interacting with clinical and ecological aspects should be conducted in order to create a more reliable distribution map of this important systemic mycosis and its etiological agents. In addition, higher throughput sequencing methods should be addressed in order to get a better resolution of *Paracoccidioides* species diversity and distribution.

## Supporting Information

S1 Table Sequences obtained from environmental *amplicon* with ITS1;5.8S;ITS2 partial sequences deposited in the *Genbank* platform from the NCBI database. (DOC)

S1 Fig Imagens of agarose gel electrophoresis by molecular detection of ITS1;5.8S;ITS2 of *Paracoccidioides* spp. in soil and aerosol samples of RO, GO and MG states of Brazil. Images (A, B, C, D, E and F).
(DOC)
