# Correction: Environmental Mapping of Paracoccidioides spp. in Brazil Reveals New Clues into Genetic Diversity, Biogeography and Wild Host Association

**DOI:** 10.1371/journal.pntd.0004692

**Published:** 2016-04-29

**Authors:** Thales Domingos Arantes, Raquel Cordeiro Theodoro, Marcus de Melo Teixeira, Sandra de Moraes Gimenes Bosco, Eduardo Bagagli

The captions for Figs 3 and 4 are incorrectly switched. The caption that is attached to Fig 3 should be attached to Fig 4, and the caption that is attached to Fig 4 should be attached to Fig 3. The figure images appear in the correct order.

**Fig 3:**
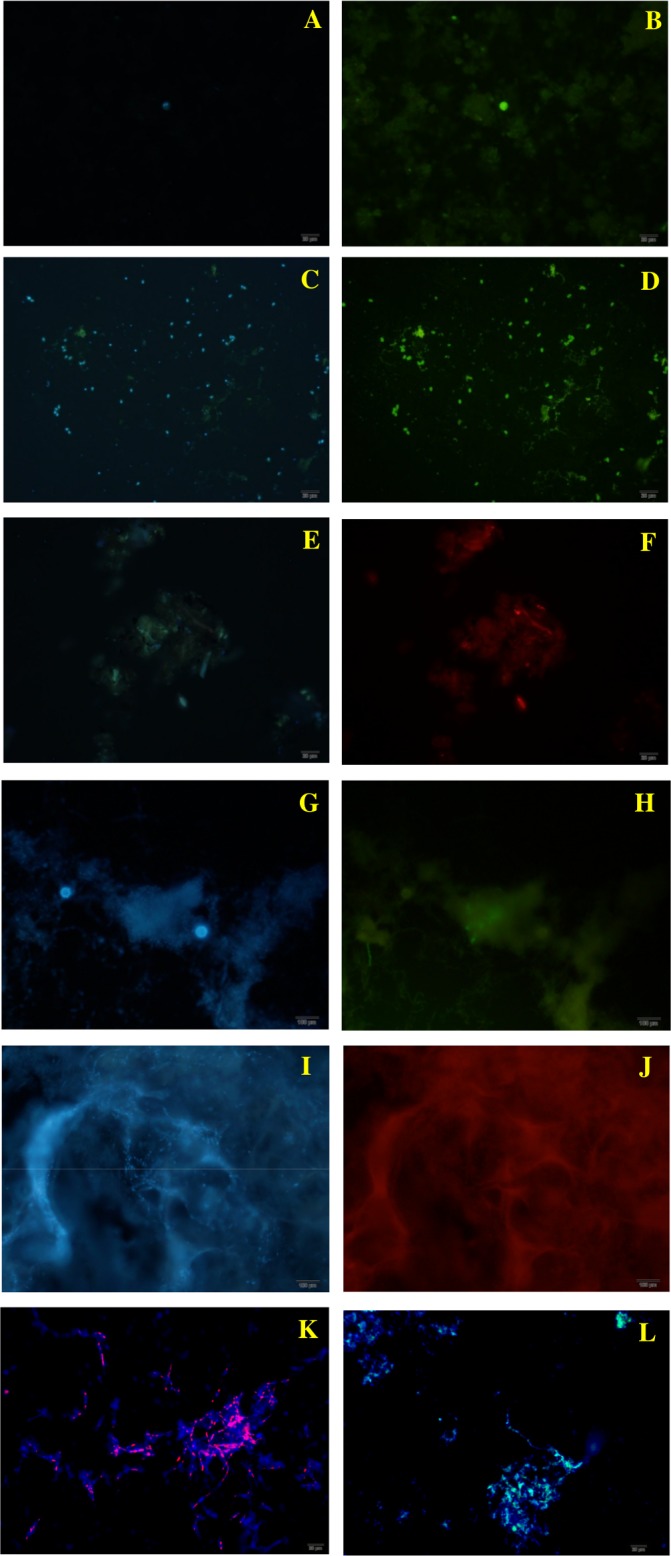
Fungal structures (400X) visualized by FISH and TSA-FISH techniques for aerosol samples and controls. A: aerosol samples from Goiás with DAPI. B: aerosol samples from Goiás with P. brasiliensis probe. C: aerosol sample from Rondônia, with DAPI. D: aerosol samples from Rondônia with P. brasiliensis probes. E: aerosol sample from Goiás with DAPI. F: aerosol sample from Goiás with P. lutzii probe. G and I: Histoplasma capsulatum with DAPI. H: Histoplasma capsulatum with P. brasiliensis probe (specificity control). J: Histoplasma capsulatum with P. lutzii probe (specificity control). K: isolate Pb01 (P. lutzii) with P. lutzii probe (positive control). L: isolate T16B1 (P. brasiliensis) with P. brasiliensis probe. The probe for P. brasiliensis is conjugated with Horseradish Peroxidase/HRP and P. lutzii probe is labeled with Texas Red/TXR, and genetic material is labeled with DAPI.

**Fig 4:**
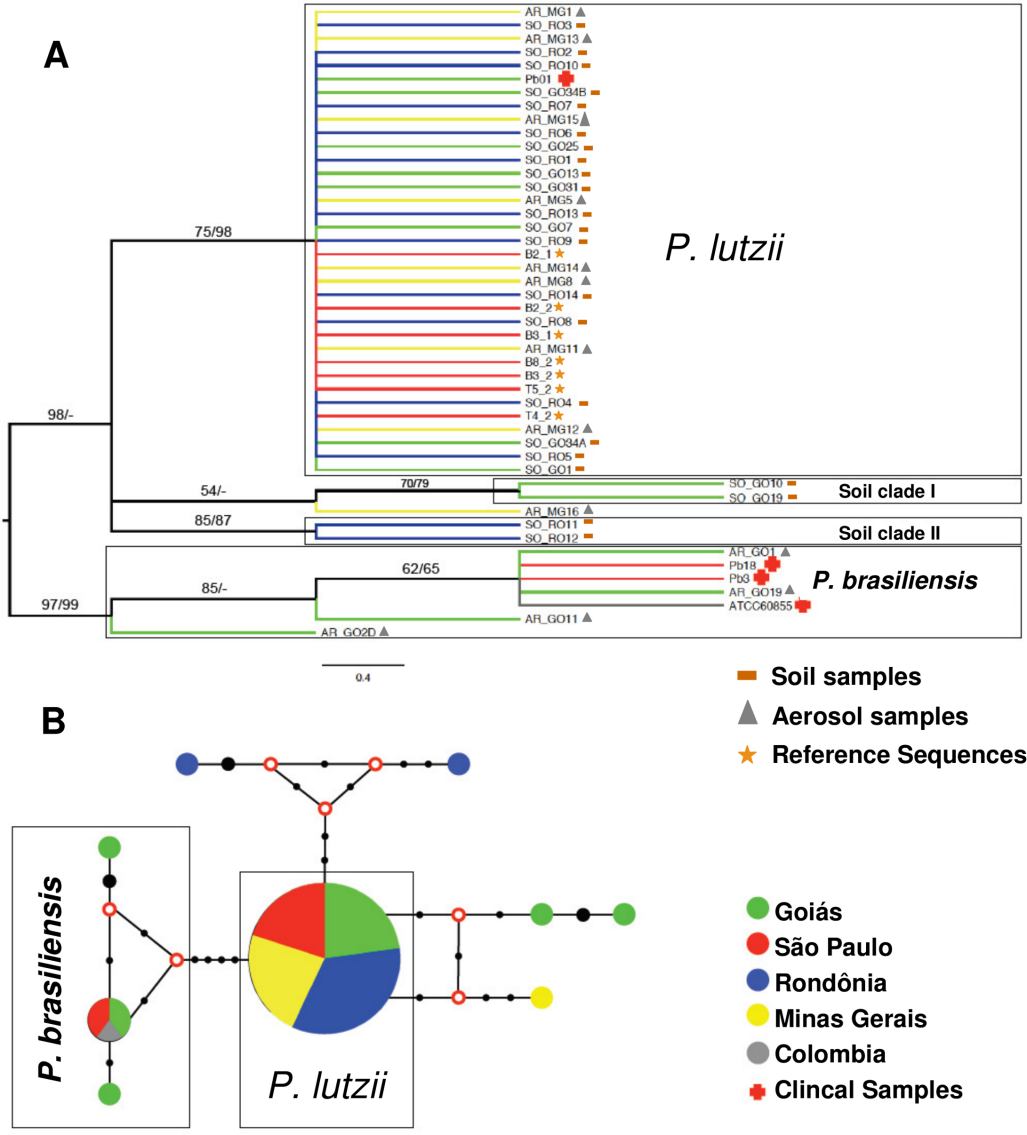
A) Molecular Phylogenetic Analysis by of ITS locus revealed by Maximum Likelihood and Neighbor Joining methods, using the Jukes-Cantor model parameters with range correction. Replication percentages on the tree are grouped in the bootstrap test (1000 replicates) and shown next to the branches. The sequences related to environmental samples are identified by acronyms SO_GO (Soil of Goiás) and AR_GO (Aerosol Goiás), AR_MG (Aerosol Minas Gerais) and SO_RO (Soil of Rondônia). B) Median-joining network showing the unique haplotypes of the Soil Clades I and II. Circles are proportional to haplotype frequency and numbers of mutations are represented by black dots. Red circles represent hypothetical missing intermediates (median vectors).
