# Supplementary material for: Environmental Mapping of Paracoccidioides spp. in Brazil Reveals New Clues into Genetic Diversity, Biogeography and Wild Host Association
Source: PLoS Negl Trop Dis. 2016 Apr 5;10(4):e0004606. doi: 10.1371/journal.pntd.0004606 (PMC4821608; doi:10.1371/journal.pntd.0004606)
Supplement: S1 Table — (DOC) [file pntd.0004606.s001.doc]

**Supporting Information – S1**

**S1 Table. Sequences obtained from environmental *amplicon* with ITS1;5.8S;ITS2 partial sequences deposited in the Genbank platform from the NCBI database**.

| **Sequence Number** | **Access Number** | ***Amplicon* Size (bp)** | **Definition of Amplicon** |
| --- | --- | --- | --- |
| **01** | **KP636439** | **372** | Uncultured *Paracoccidioides* clone SO_GO1_soil |
| **02** | **KP636440** | **380** | Uncultured *Paracoccidioides* clone SO_GO7_soil |
| **03** | **KP636441** | **334** | Uncultured *Paracoccidioides* clone SO_GO10_soil |
| **04** | **KP636442** | **376** | Uncultured *Paracoccidioides* clone SO_GO13_soil |
| **05** | **KP636443** | **330** | Uncultured *Paracoccidioides* clone SO_GO19_soil |
| **06** | **KP636444** | **389** | Uncultured *Paracoccidioides* clone SO_GO25_soil |
| **07** | **KP636445** | **390** | Uncultured *Paracoccidioides* clone SO_GO31_soil |
| **08** | **KP636446** | **394** | Uncultured *Paracoccidioides* clone SO_GO34A_soil |
| **09** | **KP636447** | **375** | Uncultured *Paracoccidioides* clone SO_GO34B_soil |
| **10** | **KP636448** | **336** | Uncultured *Paracoccidioides* clone AR_GO1_aerosol |
| **11** | **KP636449** | **359** | Uncultured *Paracoccidioides* clone AR_GO2D_aerosol |
| **12** | **KP636450** | **388** | Uncultured *Paracoccidioides* clone AR_GO11_aerosol |
| **13** | **KP636451** | **393** | Uncultured *Paracoccidioides* clone AR_GO19_aerosol |
| **14** | **KP636452** | **393** | Uncultured *Paracoccidioides* clone AR_MG1_aerosol |
| **15** | **KP636453** | **388** | Uncultured *Paracoccidioides* clone AR_MG5_aerosol |
| **16** | **KP636454** | **394** | Uncultured *Paracoccidioides* clone AR_MG8_aerosol |
| **17** | **KP636455** | **395** | Uncultured *Paracoccidioides* clone AR_MG11_aerosol |
| **18** | **KP636456** | **392** | Uncultured *Paracoccidioides* clone AR_MG12_aerosol |
| **19** | **KP636457** | **392** | Uncultured *Paracoccidioides* clone AR_MG13_aerosol |
| **20** | **KP636458** | **395** | Uncultured *Paracoccidioides* clone AR_MG14_aerosol |
| **21** | **KP636459** | **394** | Uncultured *Paracoccidioides* clone AR_MG15_aerosol |
| **22** | **KP636460** | **393** | Uncultured *Paracoccidioides* clone AR_MG16_aerosol |
| **23** | **KP636461** | **397** | Uncultured *Paracoccidioides* clone SO_RO1_soil |
| **24** | **KP636462** | **391** | Uncultured *Paracoccidioides* clone SO_RO2_soil |
| **25** | **KP636463** | **391** | Uncultured *Paracoccidioides* clone SO_RO3_soil |
| **26** | **KP636464** | **389** | Uncultured *Paracoccidioides* clone SO_RO4_soil |
| **27** | **KP636465** | **389** | Uncultured *Paracoccidioides* clone SO_RO5_soil |
| **28** | **KP636466** | **387** | Uncultured *Paracoccidioides* clone SO_RO6_soil |
| **29** | **KP636467** | **390** | Uncultured *Paracoccidioides* clone SO_RO7_soil |
| **30** | **KP636468** | **389** | Uncultured *Paracoccidioides* clone SO_RO8_soil |
| **31** | **KP636469** | **391** | Uncultured *Paracoccidioides* clone SO_RO9_soil |
| **32** | **KP636470** | **389** | Uncultured *Paracoccidioides* clone SO_RO10_soil |
| **33** | **KP636471** | **389** | Uncultured *Paracoccidioides* clone SO_RO11_soil |
| **34** | **KP636472** | **396** | Uncultured *Paracoccidioides* clone SO_RO12_soil |
| **35** | **KP636473** | **390** | Uncultured *Paracoccidioides* clone SO_RO13_soil |
| **36** | **KP636474** | **389** | Uncultured *Paracoccidioides* clone SO_RO14_soil |
| **Total # of nucleotide sequences** | | | **36** |

**Legend:** SO = soil sample from armadillo burrows; AR = aerosol samples from cyclonic aerosol sampler (NIOSH) in armadillo burrows; RO = Rondônia state; GO = Goiás state; MG = Minas Gerais state; (bp) Base pairs.
