# Supplementary material for: Environmental Mapping of Paracoccidioides spp. in Brazil Reveals New Clues into Genetic Diversity, Biogeography and Wild Host Association
Source: PLoS Negl Trop Dis. 2016 Apr 5;10(4):e0004606. doi: 10.1371/journal.pntd.0004606 (PMC4821608; doi:10.1371/journal.pntd.0004606)
Supplement: S1 Fig — Images (A, B, C, D, E and F). (DOC) [file pntd.0004606.s002.doc]

**Supporting Information – S2**

**S2 Figure.** Imagens of agarose gel electrophoresis by Nested PCR to ITS1;5.8S;ITS2 of *Paracoccidioides* spp. in soil and aerosol samples of RO, GO and MG states of Brazil. Images (A, B, C, D, E and F).

**
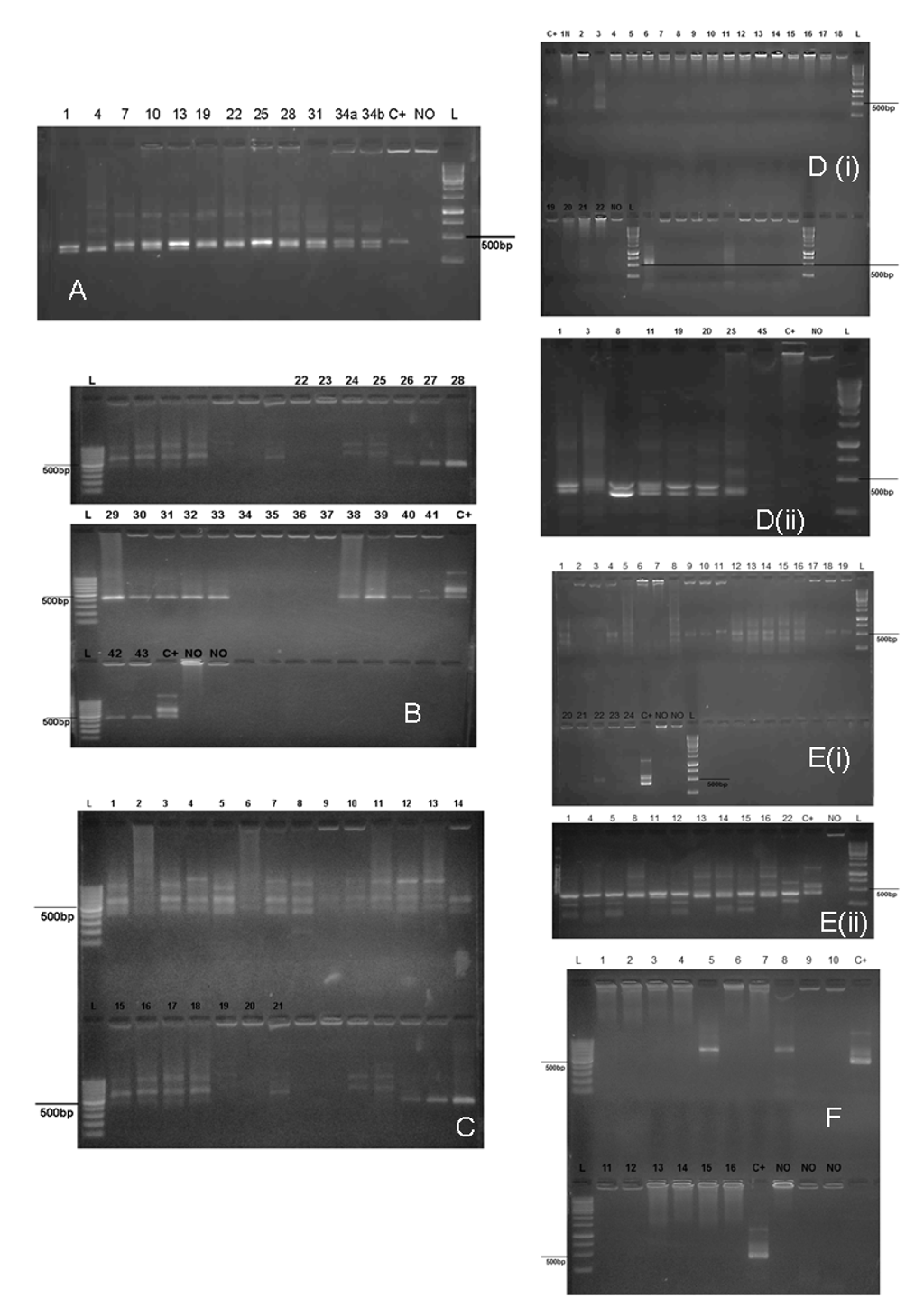
**

**S2 Legend:**

**Image A.** Agarose gel electrophoresis at 1.5% w/v of Nested PCR (ITS region) from GO soil samples (1-34b), demonstrated positive *amplicons* with approximately 450bp, positive control of *Paracoccidioides* spp. DNA (C +), negative control reaction (NO) and 100bp marker (L).

**Images B and C. (B)** Agarose gel electrophoresis at 1.5% w/v of Nested PCR (ITS region) from RO soil samples (22-43), demonstrated positive *amplicons* with approximately 450bp, positive control of *Paracoccidioides* spp. DNA (C +), negative control reaction (NO) and 100bp marker (L). **(C)** Agarose gel electrophoresis at 1.5% w/v of Nested PCR (ITS region) from “Dark Earth” soil samples (1-21), demonstrated positive *amplicons* with approximately 450bp, positive and negative control in image B, because it is the same PCR reaction controls for all samples tested in these images.

**Images D. (i)** Agarose gel electrophoresis at 1.5% w/v of Nested PCR (ITS region) from GO aerosol samples (1N-22), demonstrated positive *amplicons* with approximately 450bp, positive control of *Paracoccidioides* spp. DNA (C +), negative control reaction (NO) and 100bp marker (L). **(ii)** Concentrated samples for a new PCR reaction to confirm the first detection.

**Image E. (i)** Agarose gel electrophoresis at 1.5% w/v of Nested PCR (ITS region) from MG aerosol samples (1-24 “up” and 1-22 “down”), demonstrated positive *amplicons* with approximately 450bp, positive control of *Paracoccidioides* spp. DNA (C +), negative control reaction (NO) and 100bp marker (L). **(ii)** Concentrated samples for a new PCR reaction to confirm the first detection in the same conditions of reaction.

**Image F.** Agarose gel electrophoresis at 1.5% w/v of Nested PCR (ITS region) from RO aerosol samples (1-16), demonstrated positive *amplicons* with approximately 450bp, positive control of *Paracoccidioides* spp. DNA (C +), negative control reaction (NO) and 100bp marker (L).
